# Hydrogen Valorization from Industrial Waste Streams Using Matrimid/LaNi_5_ Mixed Matrix Hollow Fiber Membranes

**DOI:** 10.1021/acsapm.6c01367

**Published:** 2026-05-19

**Authors:** Gonzalo Moral, Alfredo Ortiz, Daniel Gorri, Inmaculada Ortiz

**Affiliations:** Department of Chemical and Biomolecular Engineering, 16761 Universidad de Cantabria, Av. Los Castros 46, 39005 Santander, Spain

**Keywords:** Hydrogen recovery, industrial waste gas streams, mixed matrix hollow fiber membranes, polymer−metal hydride membranes, mass transfer modeling

## Abstract

The valorization of industrial residual gas streams through hydrogen recovery is a key strategy for improving process efficiency and reducing environmental impact. In this context, the practical application of polymeric-based membranes, relies on enhancing the selectivity-permeability trade-off, typically achieved through the incorporation of optimized fillers, while ensuring scalability. In this work, LaNi_5_, a hydrogen storage intermetallic compound, was incorporated into a polyimide matrix to increase the membrane affinity toward H_2_ and processed in a hollow fiber configuration. The mixed matrix hollow fiber membranes were evaluated through gas permeation experiments combined with modeling under operating conditions representative of industrial residual gas streams. The use of LaNi_5_ at 5 wt %, uniformly distributed along the fibers as confirmed by SEM-EDX analysis, delivered 2.5 times higher H_2_ permeance (38.4 GPU) compared to pristine Matrimid fibers. The favorable absorption of H_2_ on LaNi_5_ resulted in enhanced selectivity with a value of 6.2 for H_2_/CO_2_ and nearly 65 for bulk compounds (H_2_/N_2_, H_2_/CH_4_ and H_2_/CO). Furthermore, the membranes exhibited stable performance over approximately 580 h of continuous operation. Finally, the model of hydrogen permeation through the hollow fiber membrane that accounts for the contribution of the polymer, and the filler showed a good agreement with the experimental data, as illustrated in the parity plot, where deviations fall within the predefined ±15% threshold. Thus, this work advances the development of high-performance membranes for hydrogen recovery from waste gas streams while supporting process scale-up through hollow fiber membrane implementation.

## Graphical abstract

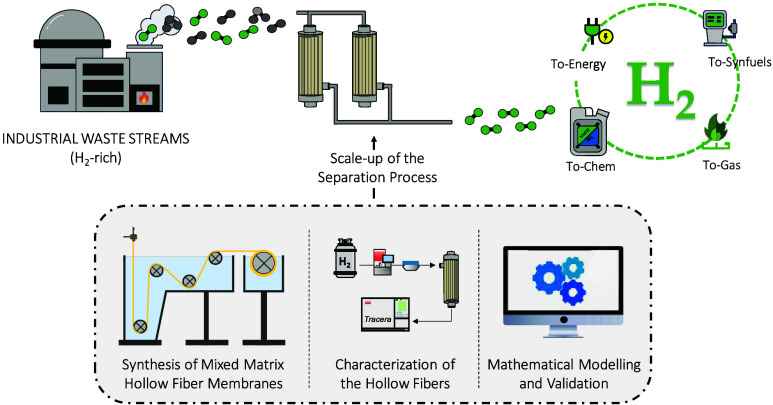

## 1 Introduction

The transition toward a sustainable and decarbonized energy model requires the development of advanced technologies capable of reducing greenhouse gas emissions while maintaining industrial competitiveness.^1,2^ In this scenario, H_2_ is expected to play a strategic role as a clean energy vector due to its high energy content and its potential to decouple energy systems from carbon-based fuels.^3^ H_2_ can be used in multiple sectors, from energy storage and grid balance to feedstock in the chemical industry and clean fuel in the transportation sector.^4,5^ However, its implementation in large-scale processes remains conditioned by the availability of efficient and cost-effective separation technologies, as conventional alternatives are highly energy-intensive processes, especially when H_2_ is obtained from low-purity sources such as industrial off-gas streams.^6^ A relevant example of these H_2_-rich waste streams includes coke oven gas (COG), ammonia purge gas (APG), or methanol purge gas (MPG), which typically contain 50–70 vol % of H_2_ along with CO_2_, N_2_, CH_4_ and CO.^7,8^ Thus, the recovery of H_2_ from these streams is aligned with circular economy principles and could significantly improve energy efficiency in sectors such as steelmaking, ammonia synthesis, or petroleum refining.^9^ Nevertheless, robust separation technologies must be designed to deliver high H_2_ purity with low energy consumption while maintaining stability under industrial conditions. In recent years, polymeric membranes have emerged as an attractive alternative to Pd-based membranes for H_2_ recovery from industrial waste streams, whereas Pd membranes are mainly employed to produce H_2_ at fuel cell grade purity.^10^ Among them, polyimides such as Matrimid 5218 have demonstrated good chemical and thermal stability, as well as promising gas separation properties.^11^ However, polymeric materials are limited by the trade-off between permeability and selectivity. To overcome this limitation, mixed matrix membranes (MMMs) have been developed by incorporating inorganic fillers into the polymer matrix, aiming to combine the processability of polymers with the molecular sieving or sorption-selective capabilities of the dispersed phase.^12^ Regarding molecular sieve mechanisms, metal organic frameworks (MOFs) such as zeolitic imidazole frameworks (ZIFs) and zeolites have been widely explored as fillers in MMMs.^13–15^ In this context, the performance of Matrimid-based MMMs incorporating different loadings of ZIF-8 and ZIF-90 was investigated in an earlier work conducted by our group.^16^ The resulting membranes exhibited a H_2_/CO_2_ selectivity of 4.3, compared to 2.1 for the pristine polymer, together with nearly doubled permeability. Similarly, Chaidou et al.^17^ investigated the effect of incorporating zeolites 13X, 4A and ZSM5 into Matrimid. The most promising results were obtained for the Matrimid/13X membrane with a loading of 15 wt % that leads to a H_2_ permeability coefficient of 32 Barrer and H_2_/CO_2_ selectivity of 2.5. Although fillers with pore sizes between the kinetic diameter of H_2_ (2.89 Å) and CO_2_ (3.3 Å), have demonstrated the potential to improve the permeability-selectivity trade-off, the high purity requirements of H_2_ use at industrial scale has led to alternative approaches. In this sense, the incorporation of metallic compounds capable of reversibly interacting with H_2_ has gained attention in recent years. As an example, in a previous study by our research group, Matrimid/LaNi_5_ MMMs were developed for H_2_ recovery from gas mixtures simulating industrial waste streams such as COG, APG, and MPG. The use of LaNi_5_, a hydrogen storage material that can reversely absorb H_2_ under mild conditions, significantly enhanced the membrane affinity, achieving a H_2_ permeability of 107 Barrer and H_2_/CO_2_ selectivity of 14.5 at 2.5 bar and 20 °C when a LaNi_5_ loading of 10 wt % was used.^18^ While these results are promising, most studies on MMMs have been limited to flat-sheet configurations, which, despite being suitable for fundamental evaluation, face limitations in scalability. In contrast, hollow fiber membranes (HFMs) offer a much higher surface area-to-volume ratio making them more suitable for industrial implementation.^19^ Nonetheless, the synthesis of defect-free mixed matrix hollow fiber membranes (MMHFMs) remains a major challenge, as most reported HFMs require an additional poly­(dimethylsiloxane) (PDMS) coating to seal minor defects. David et al.^20^ were among the first to report the use of defect-free polyimide hollow fibers for H_2_ separation. The Matrimid HFMs delivered a H_2_/CO_2_ selectivity factor ranging from 3.0 to 3.5 at 30 °C, that was comparable to flat-sheet configuration. Moreover, the optimization of spinning conditions has proven crucial to achieve defect-free structures. In this regard, Dong et al.^21^ investigated the synthesis of Matrimid HFMs for O_2_/N_2_ separation, demonstrating that the tuning of dope composition and spinning conditions could lead to HFMs with dense outer selective layers, without the need for post-treatment. On the one hand, the use of a volatile solvent in the dope solution that can evaporate during the air gap (distance from the outlet of the spinneret to the water bath) transition, results in a higher polymer concentration on the shell side and thus, increasing the thickness of the dense selective layer. Moreover, a higher ratio of solvent in the bore liquid compared to water and an intermediate air gap could also reduce the formation of macrovoids. In this context, the integration of inorganic fillers to produce MMHFMs represents a promising route to overcome current limitations and facilitate membrane scale-up. For instance, Etxeberria-Benavides et al.^22^ successfully prepared polybenzimidazole (PBI)-based MMHFMs incorporating ZIF-8 by dry-wet spinning, achieving a H_2_ permeance of 65 GPU and H_2_/CO_2_ selectivity of 18. PBI membranes find their main niche of application at elevated operating temperatures (>150 °C). Nevertheless, for practical scenarios such as H_2_ recovery from COG after the cleaning stage, it would be desirable to develop HFMs with improved performance at lower temperatures. Thus, this work aims to address the current gap between high-performance materials and scalable membrane configurations by developing and characterizing Matrimid/LaNi_5_ MMMs in HFMs. The polymer/intermetallic membrane, whose high performance has previously been validated in flat-sheet configuration, was investigated in HFMs configuration by gas permeation experiments using pure and multicomponent gas mixtures representative of industrial waste gas streams. The effect of LaNi_5_ incorporation on H_2_ permeance and selectivity was evaluated and compared to pristine Matrimid fibers. In addition, a mathematical model describing gas permeation flux through the hollow fiber module was implemented to validate the experimental data and to assess the contribution of both the polymer matrix and the filler to the total flux.

## 2 Materials and Methods

### 2.1 Chemicals

Hydrogen, nitrogen, carbon dioxide, carbon monoxide, methane and helium were supplied by Air Liquide with a purity of 99.5 vol %. Matrimid 5218 was kindly provided by Huntsman Advanced Materials. Dichloromethane (DCM) and *N*-Methyl-2-pyrrolidone (NMP) > 99.8% and LaNi_5_ intermetallic alloy (hydrogen storage grade) were purchased from Merck.

### 2.2 Synthesis of the Membranes

The flat-sheet membranes with 5 wt % of LaNi_5_ were synthesized following the method reported in previous works, using LaNi_5_ after an activation carried out by 5 cycles (30 min per cycle) of H_2_ absorption at 10 bar and vacuum using an Autoclave Engineers reactor of 50 mL.^18^ A 5 wt % filler loading was selected to ensure the fabrication of MMHFMs with a thin and defect-free dense layer, avoiding the need for post-treatment. Higher filler contents, up to 30 wt % as reported in literature, often lead to the formation of interfacial defects that require additional sealing steps, thereby limiting the scalability of the process.^23,24^ The flat-sheet membranes that had a thickness of 75 ± 2.9 μm, were prepared with the specific aim of serving as a benchmark to compare the separation performance with the HFMs. Moreover, Matrimid/LaNi_5_ MMHFMs were manufactured by the dry-wet spinning method (Figure S1 of the Supporting Information). The spinning solutions were loaded into stainless-steel syringes of 20 mL for the dope and 100 mL for the bore liquid, respectively. Both solutions flow through a dual-orifice spinneret using a Fusion 6000-X syringe pump (dope) and a KDS 410 Legacy pump from KD Scientific (bore fluid). The nascent fibers contacted the air before being immersed into the coagulation bath and collected in a rotating wheel. The spinning parameters, summarized in Table S1, were established based on previously reported protocols.^25,26^ The dope solution was prepared under mechanical agitation at 60 °C to ensure complete dissolution of the polymer in NMP, while LaNi_5_ was simultaneously suspended in the solvent under ultrasonic treatment to promote homogeneous dispersion. Subsequently, the LaNi_5_ suspension was added to the polymer solution, and the resulting mixture was left to rest overnight for degassing. After spinning, the HFMs were immersed in a water bath for 72 h to complete the phase inversion process, followed by sequential washing in three methanol baths (20 min each) and drying at ambient conditions for 48 h.

### 2.3 Characterization of Matrimid/LaNi_5_ MMHFMs

The morphology of LaNi_5_ particles and Matrimid/LaNi_5_ MMHFMs was examined by scanning electron microscopy (SEM) using a Zeiss EVO MA 15 instrument. On the one hand, the LaNi_5_ particles were dispersed in DCM to minimize aggregation and subsequently deposited onto the sample holder, allowing the solvent to evaporate. In the case of MMHFMs, the samples were fractured in liquid nitrogen to preserve structural integrity and subsequently sputter-coated with a thin gold layer. Additionally, elemental distribution of La in the MMHFMs was analyzed by energy-dispersive X-ray spectroscopy (EDX) employing a 10 mm^2^ silicon drift detector (Oxford Instruments, model X-act) operated with INCA software. Infrared spectra of LaNi_5_ particles and Matrimid/LaNi_5_ were performed on a PerkinElmer spectrum 65 Fourier Transform Infrared Spectrometer, scanning in the 500–3000 cm^–1^ wavelength range.^27^ The measurements were carried out with a resolution of 4 cm^–1^, 150 scans and a step speed of 2 mm s^–1^. Finally, the mechanical properties were evaluated using a servo hydraulic universal testing machine equipped with a 500 N load cell and a ±50 mm actuator, employing flat pneumatic grips to ensure proper sample (125 mm length) fixation and alignment during testing. The specimens were prepared with a length of 125 mm and tested under controlled conditions at 0.5 mm s^–1^.

### 2.4 Gas Permeation Experiments

The separation performance of the membranes was studied by means of steady-state gas permeation experiments carried out in the experimental set up shown in Figure S2. The membrane modules were made with a shell of stainless steel (outer diameter: 1/2 of inch) of 15 cm and 5 fibers (22 cm^2^ module^–1^) that were introduced and sealed with epoxy resin. Gas flow rates were regulated using digital mass flow controllers (Bronkhorst F-201CV), with a maximum range of 0.1 L_N_ min^–1^ for all gases except H_2_ (0.2 L_N_ min^–1^). The permeate was continuously removed using a vacuum pump (Edwards XDS 5) and collected in a 50 mL stainless steel vessel equipped with a pressure transducer (Ashcroft GC-35, 0–10 bar). Feed pressure was set by needle valve and monitored with pressure transducers (Ashcroft GC-35, 0–8 bar) placed in the retentate line. Moreover, the MMHFMs modules were housed in a thermostatic chamber (Memmert Excel) to ensure isothermal operating conditions. Gas composition in feed and permeate was analyzed using a gas chromatograph (Shimadzu Tracera GC-2010) with a Barrier Ionization Discharge (BID) detector. Prior to testing, the fibers were activated by three cycles of H_2_ exposure (10 bar, 30 min) followed by vacuum to promote hydrogen sorption in the LaNi_5_ phase. Finally, the gas permeation experiments were conducted with pure gases and synthetic multicomponent mixtures with similar composition to the industrial waste streams under the operating conditions depicted in Table 1.

**Table 1:** Operating Conditions of the Gas Permeation Experiments

Parameters	Value
Feed Flow rate (mL min^–1^)^a^	100
Feed Composition	Pure gases, APG, COG, MPG
Temperature (°C)	20/30/50
Feed Pressure (bar)	1.25–8
Permeate Pressure (mbar)	1 (absolute)

a The chosen feed flow rate was used to ensure a low stage cut (<5%), which is defined as the ratio of the transmembrane gas flow to the feed flow rate.

## 3 Mathematical Modeling

In this part, the gas permeation model for the membrane module is formulated to establish the basis for process simulation. In this sense, the H_2_ flux model previously developed for flat-sheet membranes has been extended to hollow fiber configuration. The proposed model enables a more detailed analysis of the transport mechanisms by accounting the contribution of the polymeric matrix and the hydrogen-storage phase, incorporating the H_2_ sorption behavior through its corresponding isotherm. This approach provides additional insight compared to conventional effective-medium models, such as Maxwell-type descriptions, which typically assume ideal dispersion and may not fully capture specific sorption-diffusion interactions. Moreover, the mathematical description of the membrane unit considers the following assumptions:

- The module operates isothermally and at steady state.
- The feed is introduced in the shell side while the permeate circulates in the lumen side.
- The feed and permeate streams flow in cocurrent mode, ensuring a stable and well-defined driving force along the fiber length under the operating conditions considered.
- Plug-flow in both sides is assumed.
- The total pressure in the feed and permeate sides are kept constant as operating conditions.
- There is no pressure drop due to fluid dynamics HFMs morphology, and the only pressure gradient is the transmembrane pressure.

As has been mentioned, the flux of H_2_ was described in a previous work by the contribution of the polymer and the hydrogen storage material^18^

1 $$J_{H_{2}}\left(z\right)=J_{H_{2},\mathrm{polymer}}\left(z\right)+J_{H_{2},\mathrm{LaN}i_{5}}\left(z\right)$$

2 $$J_{H_{2}}\left(z\right)=D_{H_{2},polymer}\cdot \frac{{dC}_{H_{2},polymer}\left(z\right)}{L}+D_{H_{2},LaNi_{5}}\cdot \frac{{dC}_{H_{2},LaNi_{5}}\left(z\right)}{L}$$

where *D*_H_2_,polymer_ and *D*_H_2_,LaNi_5__ represent the diffusion coefficient of H_2_ in the polymer and intermetallic compound respectively and *L* is the thickness of the selective layer. The influence of temperature on the diffusion coefficients was described by an Arrhenius-type relation.

3 $$D_{H_{2},\mathrm{polymer}}=D_{H_{2},\mathrm{polymer}}^{\prime }\cdot \exp\left(-\frac{Ea_{D,\mathrm{polymer}}}{R\cdot T}\right)$$

4 $$D_{H_{2},LaNi_{5}}=D_{H_{2},LaNi_{5}}^{\prime }\cdot \exp\left(-\frac{Ea_{D,LaNi_{5}}}{R\cdot T}\right)$$

While the concentration on the permeate side can be assumed as 0 since the vacuum effect, the concentration at the feed interphase can be calculated from the sorption equilibrium, that in the case of the hydrogen storage compound is defined from the equation of the H_2_ isotherm in LaNi_5_. In this sense, hydrogen sorption in intermetallic compounds proceeds through a sequence of steps, namely molecular dissociation and absorption, followed by surface and bulk diffusion, and ultimately hydride formation. This mechanism determines the maximum sorption capacity (*q*_max_) which was described using a Langmuir-type expression that accounts for the effect of the feed pressure (*p*_eq_) and the corresponding equilibrium constant (*K*_eq_)­

5 $$C_{H_{2},polymer}^{F}=[S_{H_{2},polymer}\cdot p_{H_{2}}\cdot \frac{x_{polymer}}{22.4\cdot \rho_{polymer}}]\cdot \rho_{membrane}$$

6 $$C_{H_{2,}\mathrm{LaN}i_{5}}^{F}=\frac{q_{\max}\cdot K_{\mathrm{eq}}\cdot p_{\mathrm{eq}}^{6}}{1+K_{\mathrm{eq}}\cdot p_{\mathrm{eq}}^{6}}\cdot x_{\mathrm{LaN}i_{5}}\cdot \rho_{\mathrm{membrane}}$$

where *x*_polymer_, *x*_LaNi_5__, are the mass fraction of the polymer and LaNi_5_ respectively, ρ_polymer_ is the density of the polymer, ρ_membrane_ is the density of the membrane. The dependence of temperature has been described as follows

7 $$S_{H_{2},\mathrm{polymer}}=S_{H_{2},\mathrm{polymer}}^{\prime }\cdot \exp\left(-\frac{Ea_{S,\mathrm{polymer}}}{R\cdot T}\right)$$

8 $$\ln K_{n}=-\frac{\Delta H_{n}}{\mathrm{RT}}+\frac{\Delta S_{n}}{R}$$

Finally, the flux of CO_2_ and the rest of bulk compounds can be described by the solution-diffusion model, assuming sorption equilibrium at the interphase

9 $$J_{j}=P_{j}\cdot \left(p_{j}^{F}-p_{j}^{P}\right)$$

where subscript *j* refers to N_2_, CO_2_, CH_4_, and CO, *P*_*j*_ is the permeance and *p*_*j*_^F^ and *p*_*j*_^P^ are the feed and permeate partial pressures, respectively.

## 4 Results and Discussion

### 4.1 Characterization of the Matrimid/LaNi_5_ Hollow Fibers Membranes

#### 4.1.1 Morphology of the Mixed Matrix Hollow Fibers and LaNi_5_ Particles

First, the morphology of the LaNi_5_ particles and the symmetric hollow fibers were examined by SEM, as depicted in Figure 1.

**Figure 1:**
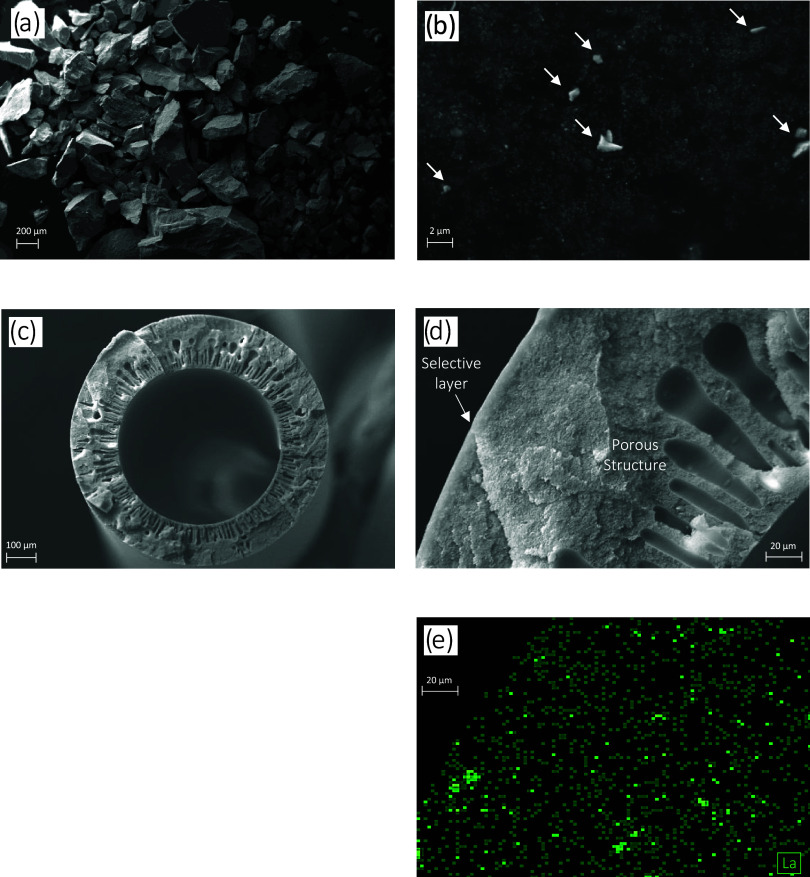
SEM images of (a) LaNi_5_ particles before activation taken from a previous work,^18^ (b) LaNi_5_ after activation, (c) Matrimid/LaNi_5_ cross section, (d) zoom of Matrimid/LaNi_5_ cross section and (e) Matrimid/LaNi_5_ EDX of the cross section showed in (c) where the green dots represent the La signal in the fiber.

As depicted in Figure 1a,b, the activation of LaNi_5_ through successive H_2_ absorption cycles led to a pronounced reduction in particle size, in agreement with previous reports.^28^ During this process, hydrogen uptake and subsequent hydride formation induce a volumetric expansion within the LaNi_5_ lattice, generating internal stresses that exceed the mechanical strength of the material and promote particle fragmentation. SEM observations from this preliminary analysis reveal a significant decrease in particle size evidencing the transformation of the initial LaNi_5_ particles into a fine powder because of the activation treatment. Regarding membranes, the MMHFMs exhibited uniform geometry with well-defined circularity and concentricity. The outer (935 μm) and inner diameters (585 μm) were determined from the average of ten independent measurements obtained from SEM micrographs using ImageJ software. In this sense, the dimensions of the MMHFMs are consistent with those previously reported for Matrimid/ZIF-8 MMHFMs, which exhibited outer and inner diameter of 915 and 570 μm, respectively.^26^ These values suggest that the addition of a filler leads to an increase in the outer diameter compared to pristine polymeric HFMs (753 and 527 μm) produced under the same spinning conditions. This behavior is likely associated with the increase in the dope solution viscosity upon filler addition, which can influence the flow dynamics during extrusion and ultimately affect fiber geometry. The cross-sectional morphology revealed an asymmetric structure, characterized by finger-like voids near the lumen side that gradually transition into a sponge-like intermediate region, finishing in a dense outer skin layer. This layered architecture is strongly influenced by the dry-wet spinning conditions. In particular, the selected air gap allowed partial evaporation of the solvent at the outer surface prior to coagulation, promoting a polymer concentration gradient that facilitates the formation of a defect-free selective layer. Moreover, the ratio between the dope and bore liquid flow rates was adjusted to ensure geometrical uniformity, while the extrusion temperature contributed to the formation of macrovoids within the substructure, thereby reinforcing the asymmetric morphology. The distribution of the inorganic filler within the fiber cross-section was evaluated by EDX mapping, as shown in Figure 1e. The elemental signal of La was homogeneously detected throughout the membrane wall, indicating good dispersion and the absence of localized agglomeration. Such uniform distribution, along with adequate interfacial compatibility between the polymer and the filler, was further confirmed by gas permeation results, which showed consistent improvements in performance. Given the relevance of the selective layer thickness in governing transport behavior in MMHFMs, its value was estimated theoretically from the measured permeability and permeance

10 $$l=\frac{permeability}{permeance}$$

where the permeability (Barrer, 1 Barrer = 3.35·10^–16^ mol mm^–2^ s^–1^ Pa^–1^) was measured from pure gas experiments at 20 °C with flat-sheet membranes and the (GPU, 1 GPU = 3.35·10^–10^ mol mm^–2^ s^–1^ Pa^–1^) from the results obtained with MMHFMs at the same operational conditions. The comparison of the values is presented in Figure S3. The spinning conditions enabled the formation of thin selective layers with measured thicknesses of 1.43 ± 0.05 μm for the different gases with values ranging from 1.37 to 1.50 μm, which may be attributed to slight irregularities in the dense selective layer along the fiber length. In this sense, the same methodology was employed by David et al.^29^ to estimate the thickness the dense selective layer of Matrimid HFMs (0.27 to 0.54 μm), reporting a similar deviation for the different gases. Moreover, the thickness of the dense selective layer in the region shown in Figure 1d was also estimated using ImageJ, yielding a comparable value (1.80 ± 0.11 μm). The slightly higher value obtained with ImageJ can be attributed to the difficulty in precisely distinguishing the transition from the sponge-like porous region to the dense selective layer, resulting in an overestimation of the mean thickness. Furthermore, the thickness of the Matrimid/LaNi_5_ MMHFMs was comparable to that reported for pristine polymeric HFMs (1.46 μm). The small reduction in the dense selective layer of MMHFMs can be attributed to the residence time of the nascent fiber in the air gap during spinning, as also observed for Matrimid/ZIF-8 MMHFMs, where the increase in the viscosity resulted in a thinner dense layer of 1.21 μm. Following the morphological characterization, the gas separation performance of the MMHFMs was evaluated under various operating conditions.

#### 4.1.2 Fourier Transform Infrared Spectroscopy Analysis

The FTIR spectra of the filler (LaNi_5_), pristine Matrimid and MMHFMs are shown in Figure 2 to assess the interactions within the composite membranes.

**Figure 2:**
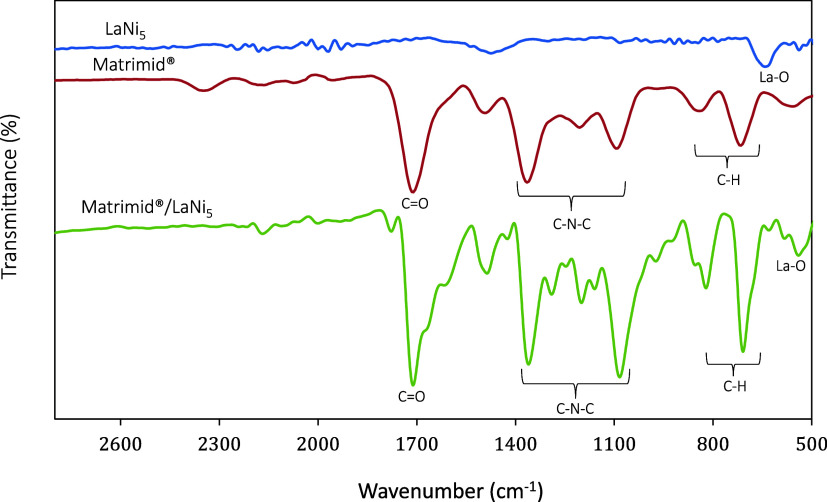
FTIR spectra of: LaNi_5_, pristine Matrimid and Matrimid/LaNi_5_ membranes. Note: the spectrum of the Matrimid membrane was taken from Moral et al.^18^

The characteristic bands of LaNi_5_ and Matrimid are consistent with those reported in the literature.^30,31^ On the one hand, the spectrum of LaNi_5_ exhibits a characteristic band around 600 cm^–1^, which can be attributed to the formation of surface oxide species due to exposure to air, both in the pristine particles and at the membrane surface, as the measurements could not be conducted under an inert atmosphere. Regarding Matrimid, the band at ∼1710 cm^–1^ is assigned to the C=O stretching of the imide groups, while the region between 1250–1290 cm^–1^ corresponds to the C–N–C stretching in the imide ring. The spectrum of the Matrimid/LaNi_5_ membrane shows the superposition of the characteristic bands of both components, confirming the successful incorporation of the filler into the polymer matrix. Although a slight shift of the LaNi_5_-related band may be observed, it is attributed to surface oxidation effects rather than to any specific interaction with the polymer. Overall, the absence of new bands or significant shifts in the characteristic peaks suggests that there is no clear evidence of new covalent bonding between the polymer and the filler, supporting a predominantly physical incorporation of LaNi_5_ within the membrane structure.

#### 4.1.3 Mechanical Stability of the Hollow Fibers

Mechanical analysis was performed to evaluate the structural stability of the hollow fiber membranes. In this sense, the parameters derived from the stress–strain curves (Figure S4, Supporting Information) are summarized in Table 2.

**Table 2:** Mechanical Stability Analysis of the Hollow Fibers

Hollow fiber	Young’s Modulus (GPa)	Ultimate tensile stress (MPa)	Strain at peak load (%)
Matrimid	1.5	39	8.8
Matrimid/LaNi_5_	0.6	19	6.4

As it can be seen in Table 2, the mechanical properties of the membranes reveal a clear effect of LaNi_5_ incorporation on the structural performance of the hollow fibers. The pristine Matrimid membrane exhibited a Young’s modulus of 1.5 GPa, an ultimate tensile strength of 39 MPa, and a strain at maximum load of 8.8%, which are in good agreement with values typically reported in the literature for Matrimid-based systems, with Young’s modulus and tensile strength commonly ranging between 1.2–2.0 GPa and 30–60 MPa, respectively.^32,33^ Upon incorporation of LaNi_5_, a significant reduction in mechanical performance was observed, with decreases of approximately 60% in Young’s modulus, 50% in ultimate tensile strength, and around 25% in strain at maximum load compared to the pristine polymer. This behavior can be attributed to the introduction of inorganic particles, which may induce local stress concentration points and disrupt the continuity of the polymer matrix, leading to a decrease in the ductility of the hollow fiber. Despite this reduction, the obtained mechanical properties remain compatible with handling and operation in hollow fiber configurations. In this regard, commercial polyimide-based modules, such as those developed by UBE, are reported to operate at pressures up to 2.4 MPa.^34^ For the industrial waste gas streams considered in this work, coke oven gas (COG) is typically available at around 20 bar, while mixed process gas (MPG) and ammonia purge gas (APG) operate at pressures above 70 bar and temperatures below 30 °C.^7^ Therefore, taking into account the measured tensile stress (∼19 MPa), the developed membranes may be considered potentially suitable for operation under these conditions, suggesting that the incorporation of LaNi_5_, while affecting mechanical resistance, does not appear to compromise the structural integrity required for practical operation.

### 4.2 Gas Permeation Experiments

The separation performance of Matrimid/LaNi_5_ MMHFMs was assessed through a series of H_2_ permeation experiments using both pure and mixed gas feeds. The effective permeance was determined by normalizing the experimental gas permeation flux with respect to the partial pressure gradient. Then, selectivity values were obtained as the ratio between the permeance of the gas pair under evaluation

11 $$P_{j}=\frac{J_{j}}{p_{j}^{F}-p_{j}^{P}}$$

12 $$\alpha_{H_{2}/i}=\frac{P_{H_{2}}}{P_{j}}$$

where subscript *j* refers to H_2_, N_2_, CO_2_, CH_4_ and CO. Since the permeate side is under vacuum, the partial pressures at that side are negligible and therefore, the driving force for permeation depends only on the feed partial pressures. All experiments were conducted in duplicate to confirm reproducibility, with deviations remaining below 5%.

#### 4.2.1 Comparison of Separation Performance between Hollow Fiber and Flat-Sheet Membranes

Initially, pure gas tests were conducted to establish a reference for comparison with multicomponent mixtures and with previously reported data for flat-sheet configurations (see Table 3). These measurements were performed at varying feed pressures, while the effects of temperature and feed composition was investigated through multicomponent gas.

**Table 3:** Ideal Selectivity Data for Matrimid and Matrimid/LaNi_5_ HFMs for Pure Gas Permeation at 20 °C and Partial Pressure Gradient of 2.5 bar^a^

Configuration	Membrane	H_2_/CO_2_	H_2_/N_2_	H_2_/CH_4_	H_2_/CO
Hollow fiber	Matrimid/LaNi_5_	6.2	62.7	60.6	67.6
Flat-sheet	Matrimid/LaNi_5_	6.3	65.9	64.0	66.2
Hollow fiber	Matrimid	2.1	38.3	32.0	36.5

a The selectivity values of Matrimid in HFMs configuration have been taken from Moral et al.^26^

As presented in Table 3, the incorporation of LaNi_5_ into the polymer matrix led to a marked improvement in selectivity. In particular, the H_2_/CO_2_ that is the most concerning gas pair in polymeric-based membranes, increased from 2.0 to 6.3 upon the addition of 5 wt % LaNi_5_. This enhancement is attributed to the hydrogen storage properties of LaNi_5_, which promotes a favorable absorption of H_2_, thereby increasing the driving force for its permeation. Gas transport in polymeric membranes typically follows the solution-diffusion mechanism, in which gas molecules are first sorbed onto the membrane surface, diffuse through the polymer matrix, and finally desorb at the permeate side. However, the incorporation of inorganic fillers introduces two additional effects. On the one hand, the inclusion of the filler between the polymer chains can disrupt the chain packing, leading to an increase in the free volume within the polymer matrix. On the other hand, depending on the intrinsic properties of the filler, an additional transport phenomenon could be induced. While fillers with small and well-defined pore sizes (i.e., MOFs or zeolites) promote molecular sieving, intermetallic compounds such as LaNi_5_ enable different diffusion pathways due to reversible H_2_ sorption.^35^ In this context, LaNi_5_ can absorb up to 1.4 wt % of H_2_, which leads to a significantly higher affinity for H_2_ compared to other gases.^36^ The comparison of ideal selectivity between flat-sheet and MMHFMs provides valuable insight into the selective layer, as any deviation from flat-sheet performance may indicate the presence of minor defects. In this study, the similar selectivity values obtained for both configurations confirmed the absence of structural imperfections in the MMHFMs, validating their effective fabrication.

#### 4.2.2 Influence of Feed Pressure and Temperature in the Permeation Flux

Moreover, since the H_2_ production is also a performance parameter of membranes, the comparison of the flux for pure and multicomponent gas mixtures is shown in Figure 3 for H_2_ and Figure S5 in the Supporting Information for the other gases.

**Figure 3:**
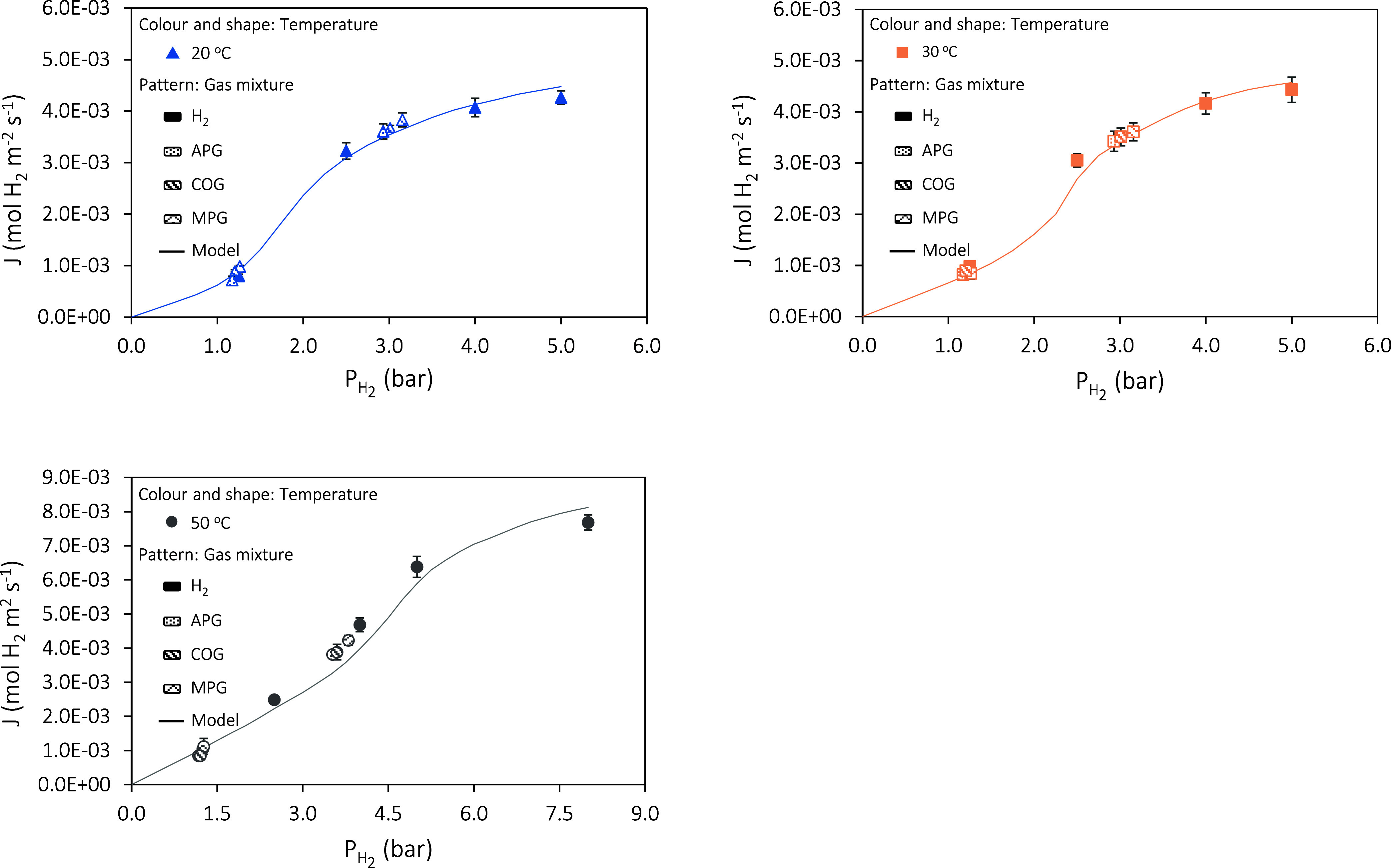
Hydrogen flux for different feed compositions at 20, 30, 50 °C.

As shown in Figure 3, the variation of H_2_ flux with feed pressure for different gas compositions through the Matrimid/LaNi_5_ MMHFMs follows a trend similar to that observed in previous works using the flat-sheet configuration, where the flux behavior correlates with the H_2_ absorption isotherms.^18^ In this regard, the flux is slightly higher than that of pristine Matrimid HFMs at low H_2_ partial pressures (<2 bar). This slight enhancement is primarily attributed to the potentially altered packing density of the polymer chains resulting from the dispersion of LaNi_5_ particles, as also observed for CO_2_ and bulk compounds, together with a minor contribution from LaNi_5_ to H_2_ sorption, which remains limited due to the low partial pressure of hydrogen under the evaluated conditions. After this initial region, the flux rises more sharply due to the higher affinity for H_2_ provided by the intermetallic filler. This behavior indicates a shift in gas transport governed by the solution-diffusion mechanism, which becomes more dominant above the so-called “plateau” pressure, suggesting a change of the diffusion pathways within the composite membrane. The “plateau” pressure represents the threshold value at which H_2_ absorption in LaNi_5_ shifts from lower to higher values. Furthermore, the “plateau” value depends on the temperature, and it has been determined to be 2.0, 3.0, and 5.5 bar at 20, 30, and 50 °C respectively.^37,38^ Thus, the H_2_ flux increases by a factor of 3, from 1.0·10^–3^ to 3.2·10^–3^ mol·m^–2^·s^–1^ as the partial pressure rises from 1.0 to 2.5 bar at 20 °C. At higher temperatures, this enhancement occurs at correspondingly higher pressures due to the increased “plateau” pressure threshold. For instance, at 50 °C, a noticeable increase in flux is observed only above 4.5 bar, reaching 7.7·10^–3^ mol·m^–2^·s^–1^ at 8 bar. In contrast, the transport behavior of CO_2_, N_2_, CH_4_, and CO (Figure S5) shows a linear dependence on the applied partial pressure difference, as predicted by the solution-diffusion model typically used to describe gas permeation through dense polymeric matrices. This linearity arises from the absence of specific interactions between these gases and the LaNi_5_ phase. Among them, CO_2_ displays the highest flux after H_2_, which is primarily associated with its high solubility coefficient in glassy polymers like Matrimid. Conversely, the fluxes of N_2_, CH_4_, and CO are lower due to their larger kinetic diameters (3.6–3.8 Å) compared to H_2_ (2.89 Å), which results in reduced diffusion coefficients.^39^ Nevertheless, the presence of LaNi_5_ increases the overall free volume of the membrane, leading to a slight enhancement in flux for these bulk components, as reported in other studies on MMMs.^40^

#### 4.2.3 Influence of Feed Pressure and Temperature in the Hydrogen Permeance and Selectivity

In this study, since the permeation flux of each gas species is governed solely by its own partial pressure gradient, independent of the presence of other components, as shown in Figures 4 and S5, the average permeance values for H_2_, CO_2_, and the bulk gases are summarized in Figure 4 and Table 4, respectively. Additional permeance and selectivity data under various operating conditions for the multicomponent mixtures are provided in Tables S2 of the Supporting Information. Despite assuming constant permeance coefficients across the different feed mixtures, the analysis of the industrial waste gas reveals a variation in H_2_ permeance, with values of 22.3, 21.6, and 18.3 GPU for COG, MPG, and APG, respectively, at feed pressure of 1.2 bar. This trend is consistent with the increasing CO_2_ content in the mixtures and can be attributed to its higher condensability and affinity toward glassy polymers. In this sense, CO_2_ sorption within the polymer matrix can induce a mild plasticization effect, increasing chain mobility and, consequently, enhancing the diffusion coefficients of the permeating species.^41^ As a result, higher CO_2_ concentrations lead to increased gas permeation, which explains the higher H_2_ permeance observed for COG and MPG compared to APG. However, this plasticization effect also reduces the diffusivity selectivity of the membrane, as the increased chain mobility diminishes the discrimination between gas species. Consequently, a decrease in selectivity toward bulk compounds is observed, with the highest coefficient obtained for the APG mixture (73.4), while it is reduced in the case of MPG to 60.3 for H_2_/N_2_ and 72.4 for H_2_/CH_4_ at 3.0 bar, 20 °C. Overall, the membrane demonstrated excellent separation performance, particularly at low temperatures, achieving average selectivity values under pure and multicomponent feed composition. Thus, these results not only addressed the H_2_/CO_2_ selectivity bottleneck of polymeric membranes but also overcome the challenge of manufacturing MMHFMs with a dense selective layer that delivers a robust separation performance across different industrial conditions.

**Figure 4:**
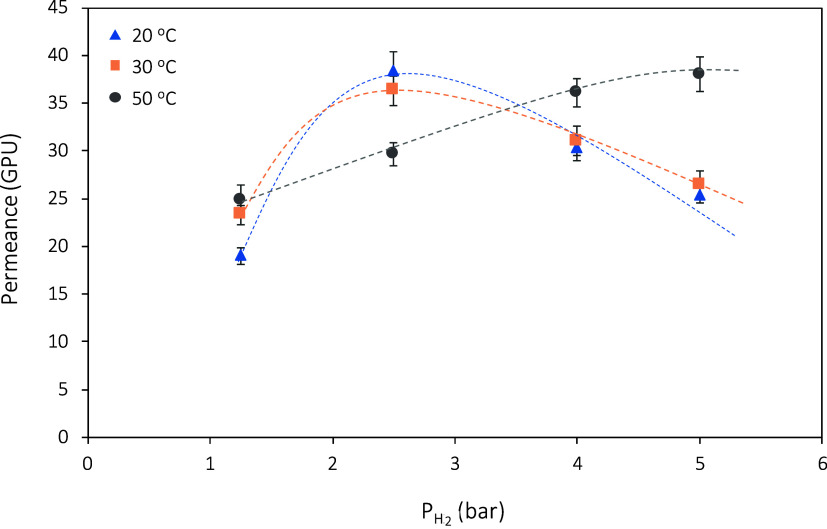
Influence of H_2_ partial pressure and temperature in H_2_ permeance. The dashed lines have been included to aid in the interpretation of the trend.

**Table 4:** Influence of Temperature on the Permeance of CO_2_ and Other Bulk Compounds

	Permeance (GPU)			
Temperature (°C)	CO_2_	N_2_	CH_4_	CO
20	6.2 ± 0.5	0.6 ± 0.1	0.6 ± 0.1	0.6 ± 0.1
30	7.8 ± 0.8	0.8 ± 0.2	0.8 ± 0.1	0.7 ± 0.2
50	11.0 ± 1.1	1.1 ± 0.1	1.0 ± 0.2	0.9 ± 0.2

Figure 4 shows that the evolution of H_2_ permeance is divided into three zones depending on the operating conditions of pressure and temperature. In the low-pressure region (below 2 bar), H_2_ transport is primarily governed by the polymer matrix, as the contribution of LaNi_5_ to H_2_ uptake is minimal under these conditions for every temperature. The observed slight increase in permeance reaching a value of 23.3 GPU at 30 °C compared to 17 GPU for pristine Matrimid is attributed to the enhanced free volume within the polymer network due to the incorporation of LaNi_5_ particles, consistent with the behavior reported in other MMHFMs.^42–44^ In the intermediate pressure range (2–3 bar), a substantial rise in H_2_ permeance is observed, driven by the H_2_ sorption properties of LaNi_5_, as the filler increases the affinity of the membrane toward H_2_. This effect is particularly evident at 20 °C, where a maximum permeance of 38.4 GPU is delivered at 2.5 bar, corresponding with maximum H_2_ absorption according to the reference “plateau” pressure at 20 °C (2 bar). Although higher temperatures enhance gas diffusivity, the H_2_ absorption simultaneously shifted to higher pressures (>3 bar), where the H_2_ enhancement in flux is less pronounced compared to the increase driven by the applied pressure gradient as can be seen in Figure 4. As a result, the highest permeance is observed at 20 °C, where despite the lower diffusivity of H_2_, it is confirmed that the transport mechanism is governed by the filler’s sorption capacity under these conditions. This shift in transport mechanism from a diffusion-controlled regime to one controlled by sorption is further supported by the modeling results presented in Section 4.3.

#### 4.2.4 Comparison of Membranes Performance

Moreover, the comparison of Matrimid/LaNi_5_ with other polymeric-based MMHFMs reported in the literature is presented in Figure 5.

**Figure 5:**
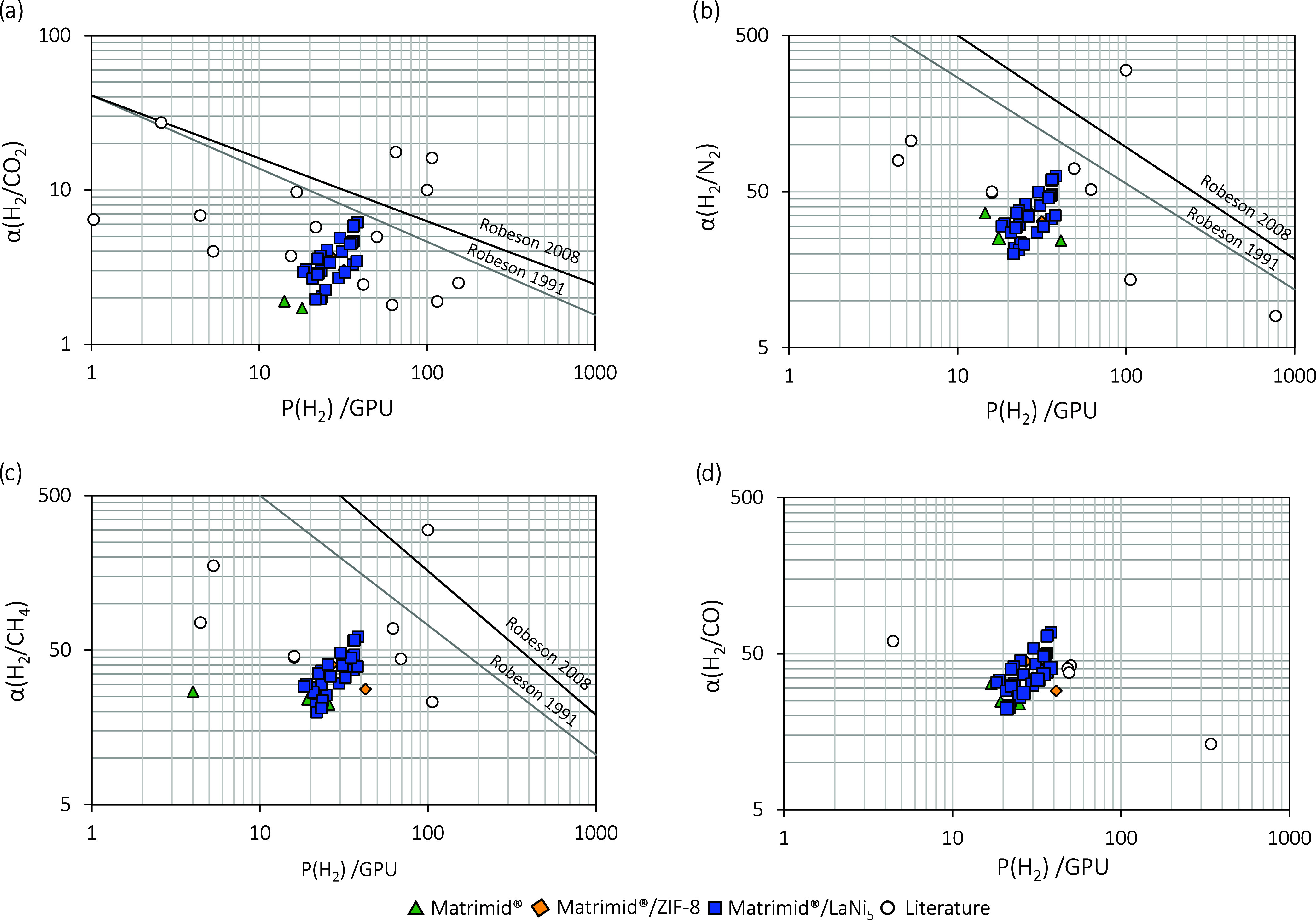
Robeson plot for polymeric-based MMHFMs: (a) H_2_/CO_2_, (b) H_2_/N_2_, (c) H_2_/CH_4_ and (d) H_2_/CO. Matrimid and Matrimid/ZIF-8 are reported from previous work^26^ while literature values were taken from refs 20,45–57 . The Robeson limits are calculated from the permeability values assuming a hollow fiber dense layer thickness of 1 μm.

Figure 5 compares the performance of the Matrimid/LaNi_5_ MMHFMs developed in this work with reported polymeric-based HFMs using Robeson plots for H_2_/CO_2_, H_2_/N_2_, H_2_/CH_4_, and H_2_/CO separations. In all cases, a noticeable improvement in both permeance and selectivity was observed compared to pristine Matrimid, which is particularly relevant considering the relatively low filler loading (5 wt %), highlighting the efficiency of LaNi_5_ as a hydrogen-selective phase. Remarkably, despite this moderate loading, the separation performance of the Matrimid/LaNi_5_ MMHFMs approached the Robeson upper bound for the most critical gas pairs, reflecting the contribution of the hydrogen sorption mechanism in mitigating the typical permeability-selectivity trade-off of polymeric membranes. Furthermore, when compared to Matrimid/ZIF-8 MMHFMs with a 5 wt % loading, previously analyzed under the same conditions, the Matrimid/LaNi_5_ MMHFMs also exhibited superior separation performance.^26^ As example, the Matrimid/LaNi_5_ MMHFMs delivered H_2_/CO_2_ selectivity of 4.7 and a value of 47 for H_2_/bulk at 2.5 bar and 30 °C, whereas Matrimid/ZIF-8 MMHFMs showed a reduction of 31% and 25% for H_2_/CO_2_ and H_2_/bulk, respectively. These results further support the advantage of hydrogen-affinity-based transport over purely molecular sieving mechanisms. Overall, the use of LaNi_5_ acting as a pathway to enhance H_2_ sorption, has demonstrated superior performance under the studied conditions compared to approaches such as polymer blending, certain polymer modification strategies and some porous fillers, when considering the same polymer matrix, whereas other modification techniques or fillers have been reported only in flat-sheet configurations.^58^ However, it should be noted that these comparisons are based on reported data obtained under different experimental conditions, and testing protocols, and therefore should be interpreted as indicative trends rather than strictly quantitative equivalences. In this sense, vapor-phase modified PBI/Matrimid reported by Lau et al.^45^ showed a lower H_2_ permeance (21.7 GPU) and slightly lower H_2_/CO_2_ selectivity (5.8) compared to HFMs developed in this work. Similarly, Torlon HFMs with surface modifications exhibited higher H_2_/CO_2_ selectivity (up to 11) but at the expense of a significant reduction in permeance (up to 7-fold), which may limit hydrogen productivity. Therefore, although surface modification improves selectivity, it comes at the expense of reduced H_2_ flux, in contrast to the incorporation of LaNi_5_, which leads to simultaneous enhancement of both permeance and selectivity. Similarly, plasma-assisted fluorination of Matrimid has been reported to improve selectivity and H_2_ permeance, however, the resulting membranes did not exhibit a remarkable increase in H_2_/CO_2_ selectivity, which remains the bottleneck in hydrogen purification.^55^ Regarding the use of porous fillers, Zulhairun et al.^56^ reported the use of titania nanotubes in PSF membranes, where the addition of 0.6 wt % led to a H_2_ permeance of 154 GPU while maintaining a H_2_/CO_2_ selectivity of 2.5, with the improvement in permeance attributed to the intrinsic volume of the nanotubes and the disruption of polymer chain packing. Moreover, the incorporation of ZIF-8 to PBI membranes was studied by Etxeberria-Benavides et al.,^22^ yielding HFMs with high H_2_ permeance (107 GPU) and H_2_/CO_2_ selectivity (16.1), operating at a temperature of 180 °C. Consistently, Kumbharkar et al.,^57^ developed PBI-based hollow fibers achieving a H_2_/CO_2_ selectivity of 27 and a H_2_ permeance of 2.6 GPU at 400 °C, further supporting the improved separation performance of PBI systems at elevated temperatures. In this context, the results of the present work highlight the suitability of Matrimid/LaNi_5_ hollow fibers for low-temperature separations, such as hydrogen recovery from industrial waste gas streams. In contrast, other combinations involving polymers with higher thermal stability can be employed for high-temperature operations approaching the range of palladium membranes. Furthermore, several studies reporting superior separation performance rely on configurations that are difficult to scale-up, such as dip-coated composite membranes or systems requiring post-treatment sealing with PDMS.^49,55^ In this sense, the fabrication of defect-free MMHFMs by a scalable dry-wet spinning method, as demonstrated in this work, underscores their potential for industrial application. In summary, the Matrimid/LaNi_5_ MMHFMs presented in this study combine moderate filler content, scalable fabrication, and operation under realistic process conditions, achieving separation performance that positions them among the most promising alternatives reported to date for H_2_ purification. In addition, stable performance was observed during permeation experiments over a total operation time of 580 h (Figure S6 in the Supporting Information). In this regard, the experimental H_2_ flux was evaluated measuring the H_2_ concentration in the permeate stream under a constant feed partial pressure of 1.2 bar, both for pure gas and multicomponent mixtures. Finally, the development of a model able to predict gas permeation through the HFMs was carried out and is reported in the next section to assist the design of separation processes based on Matrimid/LaNi_5_ MMHFMs.

### 4.3 Model of Gas Permeation through HFMs and Validation

In this study, the gas permeation model that was implemented to evaluate the contribution of hydrogen storage compounds within mixed matrix membranes in flat-sheet configuration has been updated to HFMs. Thus, the novel model is designed to be used in the simulation of the separation process using MMHFMs. In this case, the two fitting parameters: the effective preexponential factor of the diffusivity through LaNi_5_ pathway (*D*_H_2_,LaNi_5__^′^ and its associated activation energy (*Ea*_D,LaNi_5__) has been also fixed to the values estimated with the results in flat-sheet membranes. On the other hand, the permeance values for CO_2_ and the bulk components were determined experimentally using eq 11), as detailed in Table 4. The remaining physical parameters required for simulation were either experimentally measured or retrieved from prior publications and can be found in Table S3.^18^ Moreover, the simulation was conducted using Aspen Custom Modeler software and the comparison of model predictions with the experimental results across the range of tested conditions is shown in Figure 3. Furthermore, the contribution of the polymer and the LaNi_5_, were estimated under the operating conditions of the pure gas experiments to confirm the trend observed in H_2_ permeance. Table 5 details the estimated contributions of the polymer and filler to the total H_2_ flux.

**Table 5:** Estimation of Transport Contributions through Polymer and LaNi_5_ Domains to the Total H_2_ Flux

		Contribution (%)	
Temperature (°C)	*P*_H_2__ (bar)	LaNi_5_	Polymer
20	1.25	20.4	79.6
	2.5	54.1	45.9
	4	45.0	55.0
	5	36.7	63.3
30	1.25	2.5	97.5
	2.5	39.1	60.9
	4	37.8	62.2
	5	24.8	75.2
50	1.25	0.8	99.2
	2.5	5.3	94.7
	4	15.2	84.8
	5	28.4	71.6
	8	16.9	83.1

As discussed in Figure 3, the contribution of LaNi_5_ to the overall H_2_ flux is limited at low H_2_ partial pressures, accounting for only 20.4% at 1.25 bar and 20 °C. As the partial pressure increases and approaches the “plateau” region, the contribution becomes more significant, reaching a maximum of 54.1% at 2.5 bar and 20 °C. Beyond this point, the LaNi_5_ becomes saturated, and its contribution slightly decreases. Additionally, increasing the temperature reduces the relative effect of the filler on H_2_ permeation. This behavior is due to two phenomena related to the solution-diffusion and the H_2_ sorption in the intermetallic compound. On the one hand, the diffusion coefficient is enhanced at high temperatures due to the wider gap between the polymer chains leading to an increase in permeance, as it can be observed in studies of gas permeation in pristine polymeric hollow fibers.^44^ On the other hand, the “plateau” pressure required for maximum H_2_ absorption in LaNi_5_ shifts to higher values with temperature. Consequently, the contribution at 50 °C only reaches a maximum of 28.4%. Although this is remarkable considering the low LaNi_5_ loading (5 wt %), it confirms that the increase in the membrane affinity toward H_2_ is hampered by the temperature. This behavior is reflected in the permeance trend (Figure 4), where the increase in LaNi_5_ contribution from 20.4 (1.25 bar) to 54% (2.5 bar) led to an exponential increase of H_2_ permeance from 19 to 38.4 GPU. For CO_2_ and the bulk components (N_2_, CH_4_, CO), permeance values (Table 4) were calculated from experimental flux data using eq 11, with *R* ^2^ values above 0.99. Given their larger kinetic diameters and lack of interaction with LaNi_5_, the bulk compounds exhibited permeance values between 0.6 and 1.1 GPU. In contrast, CO_2_, due to its higher solubility in glassy polymers, showed permeance values between 6.2 to 11.0 GPU. Finally, although a good agreement between experimental data and model predictions can be observed in Figure 3, a parity plot was plotted (Figure 6) to further validate the proposed H_2_ flux model.

**Figure 6:**
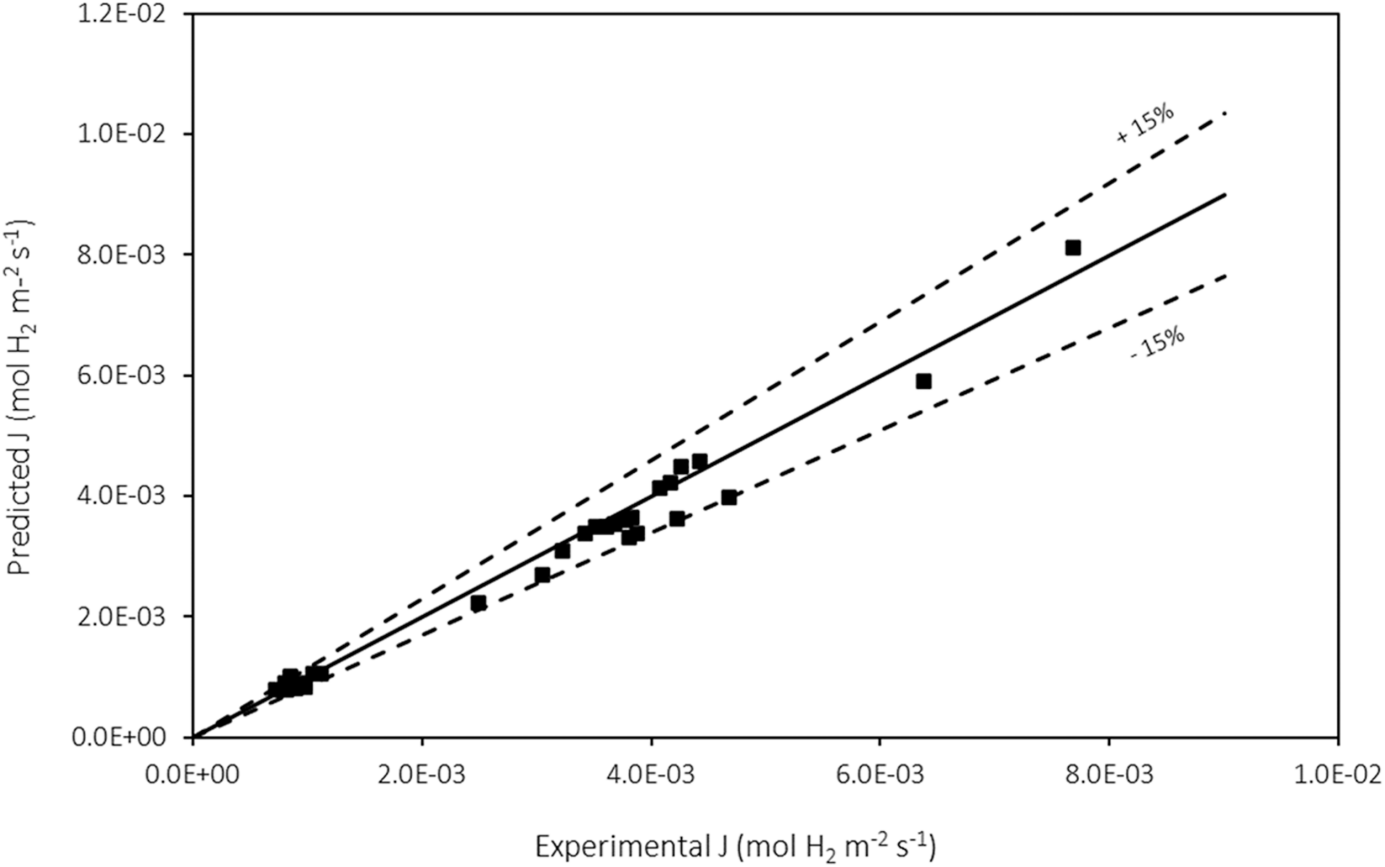
Model parity graph showing a 15% error range for H_2_ flux from pure and multicomponent mixtures at 20, 30, 50 °C.

As shown in Figure 6, most of the data points fall within 15% of acceptable error threshold. Furthermore, the lowest regression coefficient was observed for the MPG stream at 50 °C (0.92), while the average value across all operating conditions was 0.98. These results confirm that the proposed model reliably describes the H_2_ permeation behavior through a membrane incorporating hydrogen storage compounds, in both flat-sheet and HFMs configuration. This validation highlights its potential as a valuable tool for the design of real separation processes using MMHFMs incorporating intermetallic compounds as fillers.

## 5 Conclusions

This work presents the synthesis and characterization of Matrimid/LaNi_5_ MMHFMs with a 5 wt % of filler loading for hydrogen recovery from industrial waste gas streams. The fabrication of defect-free HFMs by dry-wet spinning was successfully achieved without the need for post-treatment sealing, confirming the feasibility of producing asymmetric structures with a dense selective layer. Morphological characterization through SEM and EDX confirmed not only the well-defined fiber geometry and asymmetric structure but also the homogeneous distribution of LaNi_5_ particles across the membrane thickness, ensuring a proper interfacial compatibility. The permeation tests using pure and multicomponent gas mixtures demonstrated the significant influence of LaNi_5_ on H_2_ transport. The incorporation of this hydrogen storage alloy enhanced membrane affinity toward H_2_, enabling it to surpass the conventional solution-diffusion mechanism. This behavior was especially evident under operating conditions that matched the absorption characteristics of LaNi_5_, with a maximum permeance of 38.4 GPU, H_2_/CO_2_ and H_2_/bulk selectivity of 6.2 and 63 respectively at 20 °C and 2.5 bar. Thus, the defect-free asymmetric structure with a thin dense selective layer enabled these high separation performances, as it ensured efficient gas transport while maintaining mechanical integrity. The separation performance values place the Matrimid/LaNi_5_ MMHFMs above other alternatives that use molecular sieving compounds as ZIFs, confirming their potential for high-purity H_2_ recovery. The membranes maintained stable separation performance across a range of operating conditions, including tests with multicomponent gas streams representative of coke oven gas (COG), ammonia purge gas (APG), and methanol purge gas (MPG). This robustness demonstrates their potential for reliable performance under the demanding conditions of industrial hydrogen purification processes. Moreover, the gas permeation experiments corresponded to approximately 580 h of stable operation, during which the modules exhibited consistent permeance and selectivity without signs of degradation of the transport properties. Furthermore, a gas permeation model specifically adapted to the HFMs configuration was developed and validated. By integrating the dual contribution of the polymer and the LaNi_5_ phase, the model accurately described the experimental H_2_ flux across all conditions. The successful implementation of this model highlights its relevance as a predictive tool for membrane module design and process optimization involving MMHFMs.

In conclusion, the combination of enhanced separation performance and robust operation under realistic feed conditions positions Matrimid/LaNi_5_ MMHFMs as a promising and scalable solution for hydrogen purification from industrial waste gas streams.

## Supplementary Material



## Notes

Graphical representation of hollow fiber fabrication and gas permeation setup; supplementary mechanical testing results (stress–strain curves); gas permeation data for each multicomponent mixture (PDF)
