# Supplementary material for: Hydrogen Valorization from Industrial Waste Streams Using Matrimid/LaNi5 Mixed Matrix Hollow Fiber Membranes
Source: ACS Appl Polym Mater. 2026 May 19;8(11):8810–22. doi: 10.1021/acsapm.6c01367 (PMC13274500; doi:10.1021/acsapm.6c01367)
Supplement: Supplementary file 1 [file ap6c01367_si_001.pdf]

Supporting information for: Hydrogen valorization from industrial waste streams using Matrimid®/$LaNi_5$ mixed matrix hollow fiber membranes

Authors: Gonzalo Moral[1], Alfredo Ortiz[1], Daniel Gorri[1], Inmaculada Ortiz[1*].

[1]Department of Chemical and Biomolecular Engineering. Universidad de Cantabria, Av. Los Castros 46, 39005 Santander, Spain

*corresponding author: inmaculada.ortiz@unican.es

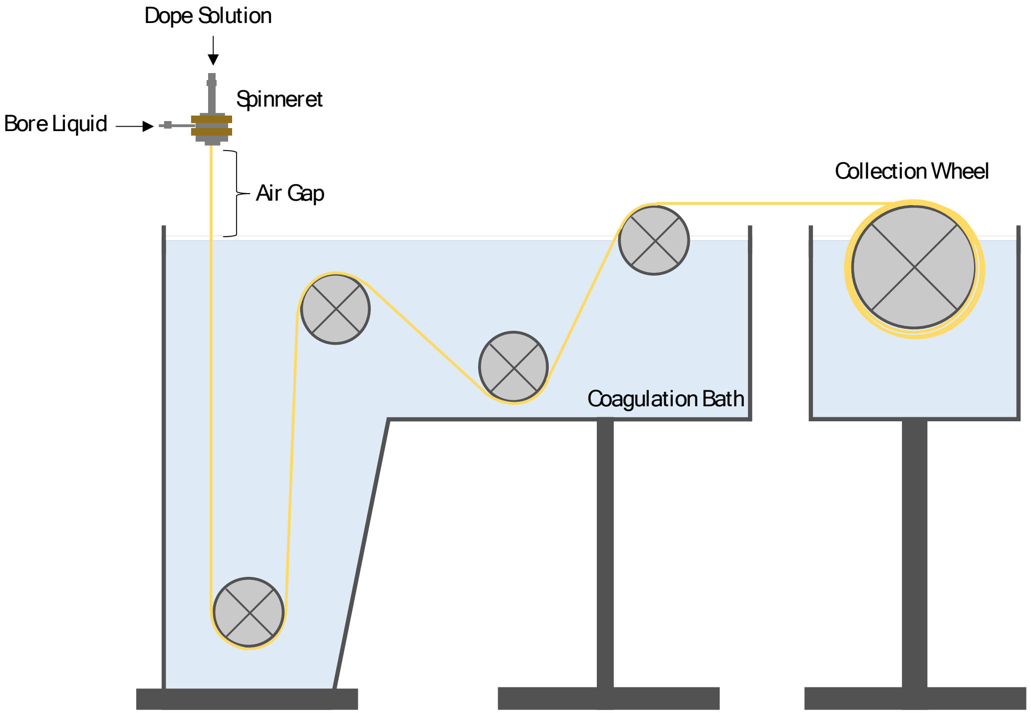

Figure S1. Illustration of the spinning process taken from previous work with permission of Elsevier[1].

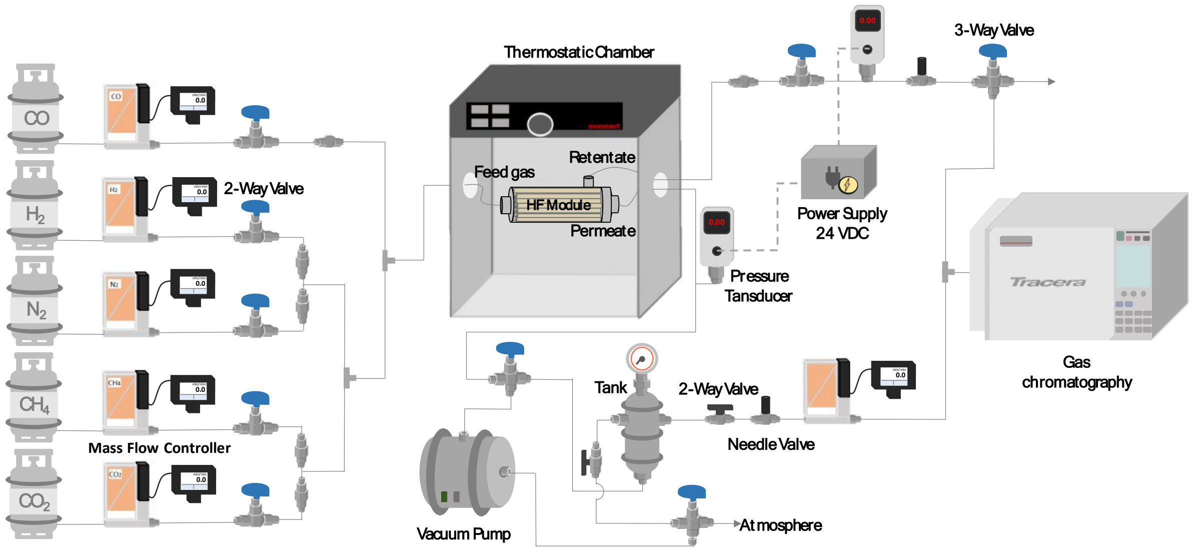

Figure S2. Schematic representation of the experimental set up for gas permeation tests.

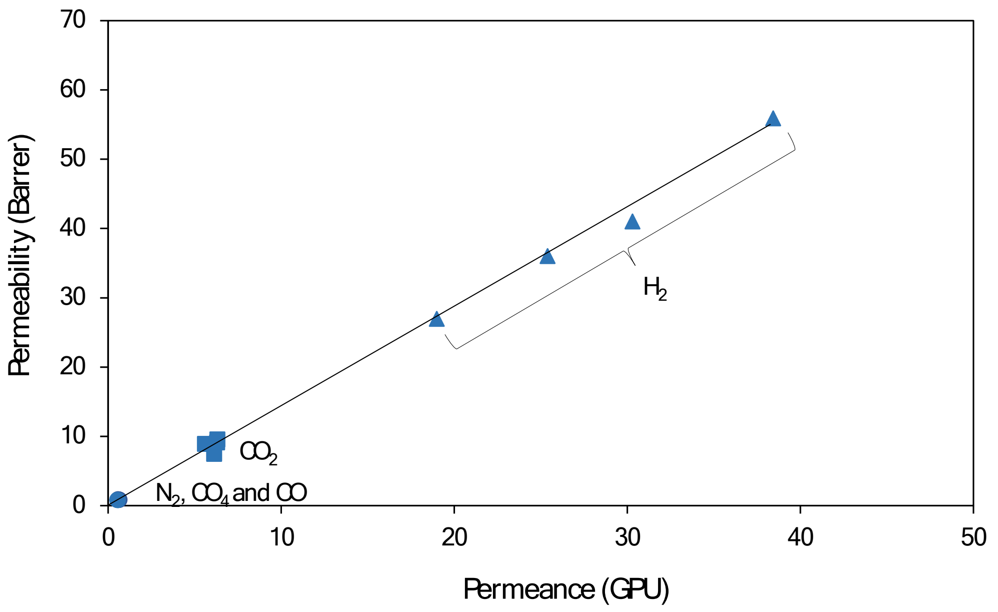

Figure S3. Permeability vs permeance for determination of the dense layer in Matrimid®/$LaNi_5$ MMHFMs. Note: For H_2, the values are shown as a function of each partial pressure, since the flux does not exhibit a linear dependence on partial pressure due to the contribution of the $LaNi_5$ in the transport.

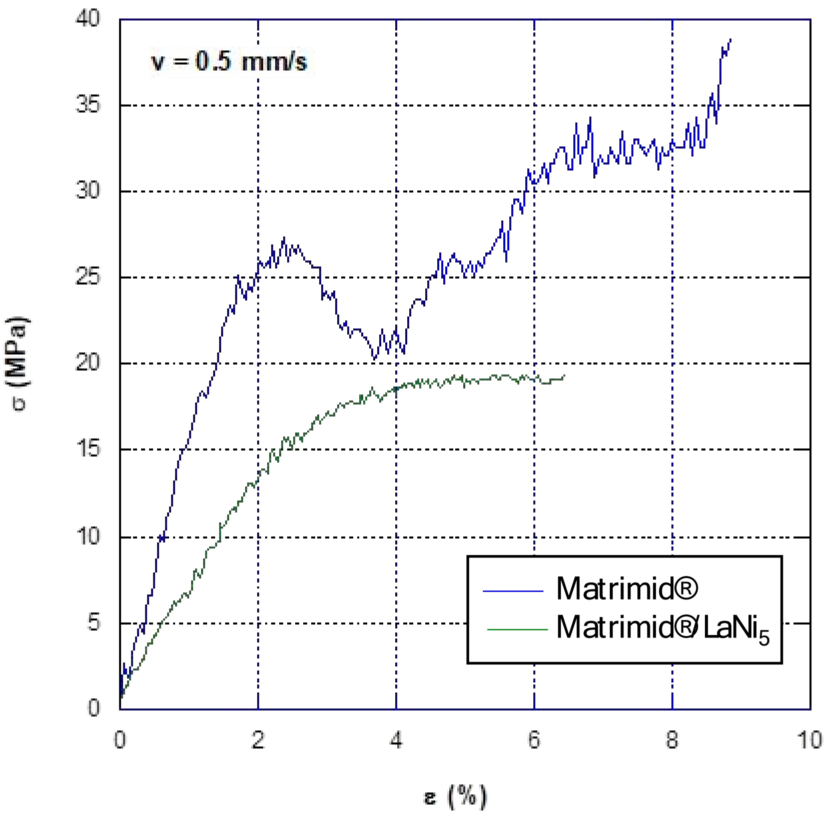

Figure S4. Comparison of stress (y-axis)-strain (x-axis) curves for pristine Matrimid® and Matrimid®/$LaNi_5$ membranes.

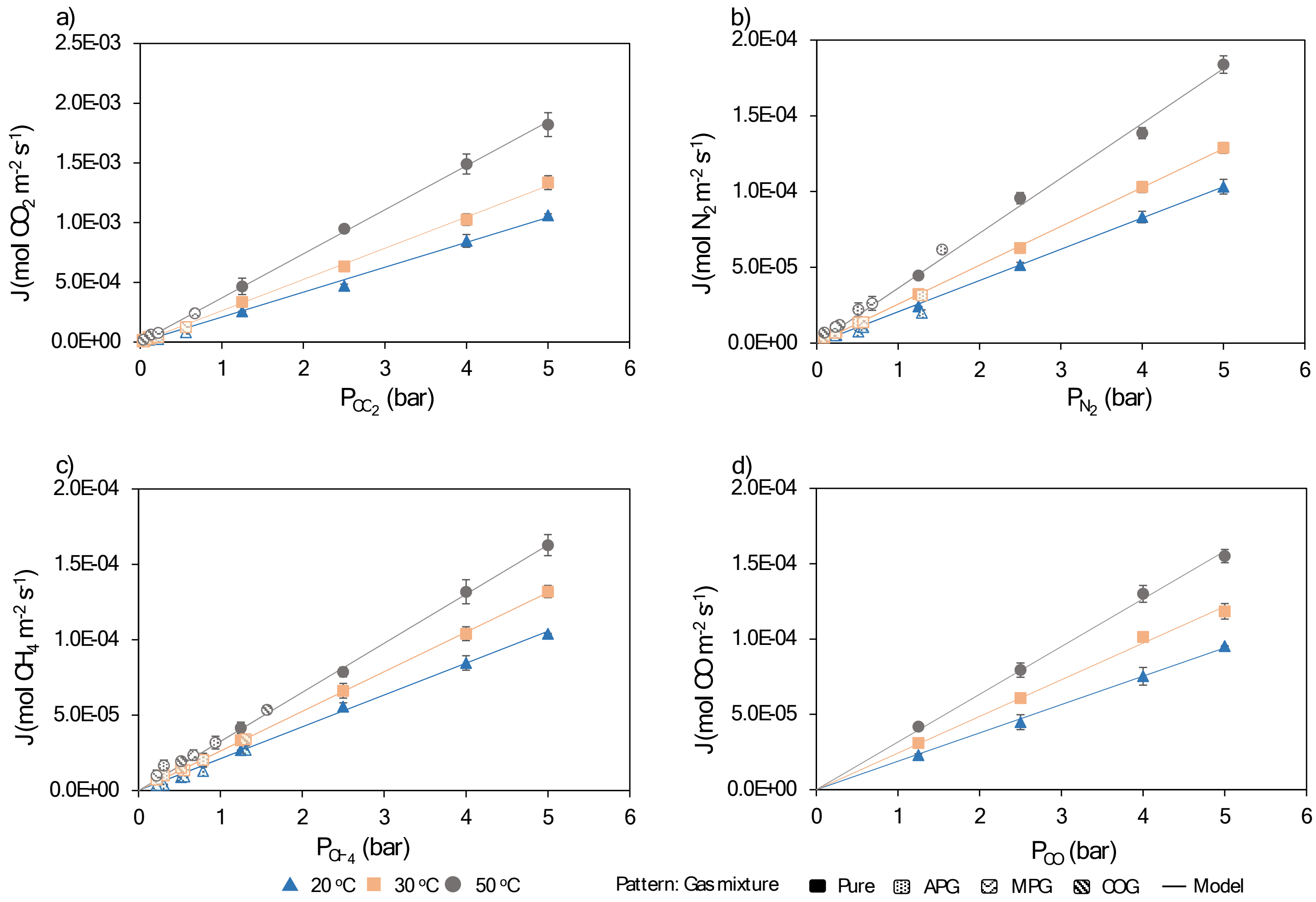

Figure S5. Flux for a) carbon dioxide, b) nitrogen, c) methane and d) carbon monoxide for the different feed streams at 20, 30, 50 °C.

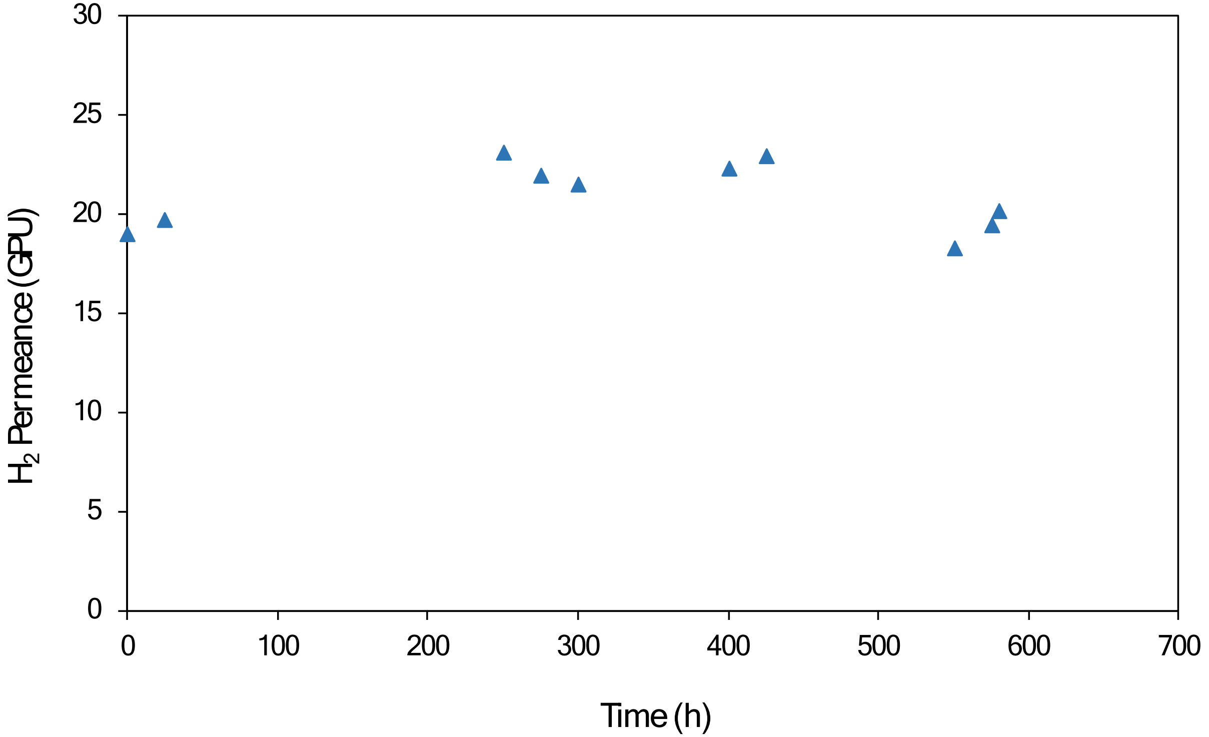

Figure S6. H_2 permeance of the Matrimid®/$LaNi_5$ mixed matrix hollow fiber membranes through the gas permeation experiments at 20 ºC and 1.2 bar of hydrogen feed partial pressure.

Table S1. Spinning conditions for the synthesis of hollow fibers.

**Operating parameters**	**Value**
Polymer content in dope solution (wt.%)	25
Filler load (wt.%)[1]	5
Dope solution flow rate (mL min^{-1})	3
Dope solution extrusion temperature (°C)	50
Bore liquid composition (wt.%)	20% NMP / 80% Water
Bore liquid flow rate (mL min^{-1})	1.5
Bore liquid extrusion temperature (°C)	Room
Air gap (cm)	16
Coagulation bath temperature (°C)	Room
Take up rate (m min^{-1})	12

[1]The filler is defined as a fraction of the polymer content.

Table S2. Gas permeances and selectivity for multicomponent mixtures in Matrimid®/$LaNi_5$ MMHFMs.

Feed	Temperature (ºC)	P_{H_2} (bar)	Permeance (GPU)				Selectivity (-)		
			H_2	CO_2	N_2	CH_4	H_2/CO_2	H_2/N_2	H_2/CH_4
COG	20	1.2	22.3 ± 0.9	5.9 ± 0.4	0.5 ± 0.1	0.6 ± 0.1	3.8	44.6	37.2
		3.0	36.4 ± 2.2				6.2	72.8	60.7
	30	1.2	22.2 ± 0.8	7.5 ± 0.7	0.7 ± 0.1	0.7 ± 0.1	3.0	31.7	31.7
		3.0	34.8 ± 3.2				4.6	49.7	49.7
	50	1.2	21.0 ± 1.2	10.6 ± 1	1.0 ± 0.2	1.0 ± 0.2	2.0	21.0	21.0
		3.6	32.0 ± 2.5				3.0	32.0	32.0
APG	20	1.2	18.3 ± 0.8	-	0.5± 0.1	0.5 ± 0.1	-	36.6	36.6
		2.9	36.7 ± 2.5					73.4	73.4
	30	1.2	20.8 ± 1.1		0.6 ± 0.1	0.8 ± 0.2		34.7	26.0
		2.9	34.8 ± 2.4					58.0	43.5
	50	1.2	21.6 ± 0.4		0.9 ± 0.1	1.0 ± 0.1		24.0	21.6
		3.5	32.3 ± 0.5					35.9	32.3
MPG	20	1.3	23.2 ± 1.0	6.0 ± 0.3	0.6 ± 0.1	0.5 ± 0.1	3.9	38.7	46.4
		3.2	36.2 ± 1.6				6.0	60.3	72.4
	30	1.3	22.5 ± 2.2	7.7 ± 0.6	0.7 ± 0.1	0.8 ± 0.1	2.9	32.1	28.1
		3.2	35.6 ± 2.3				4.6	50.9	44.5
	50	1.3	26.4 ± 2.2	10.8 ± 0.9	1.0 ± 0.2	1.0 ± 0.2	2.4	26.4	26.4
		3.8	34.7 ± 3.3				3.2	34.7	34.7

Table S3. Model parameters taken from a previous work in flat-sheet configuration.[1]

Parameter	Value
$D'_{H_2,polymer}$ $(m^2\ s^{-1})$	1.1 · 10^{-7}
$Ea_{D,polymer}$ $(kJ\ mol^{-1})$	17.3
$S'_{H_2,polymer}$ $(m^3STP\ m^{-3}bar^{-1})$	1.1 · 10^{-2}
$Ea_{S,polymer}$ $(kJ\ mol^{-1})$	-6.8
$d_{polymer}(kg\ m^{-3})$	1240
$d_{LaNi_5}(kg\ m^{-3})$	7950
$D'_{H_2,LaNi_5}$ (m^2/s)	8.7 · 10^{-11}
$Ea_{D,Lani_5}$ (kJ/mol)	6.9

Reference:

(1) Moral, G.; Ortiz, A.; Gorri, D.; Ortiz, I. Matrimid®/ZIF-8 Hollow Fiber Mixed Matrix Membranes for Hydrogen Recovery from Industrial Waste Streams. Sep. Purif. Technol. 2025, 362, 131890. https://doi.org/10.1016/j.seppur.2025.131890
